# A French classification to describe medical deserts: a multi-professional approach based on the first contact with the healthcare system

**DOI:** 10.1186/s12942-024-00366-7

**Published:** 2024-02-28

**Authors:** Marie Bonal, Cindy Padilla, Guillaume Chevillard, Véronique Lucas-Gabrielli

**Affiliations:** 1 Institute for Research and Information in Health Economics (IRDES), 75019 Paris, France; 2 Arènes-UMR 6051, RSMS-U 1309, Inserm, CNRS, EHESP, Univ Rennes, 35000 Rennes, France

**Keywords:** Accessibility, Healthcare, Classification, Medical desert

## Abstract

**Background:**

Increasing inequalities in accessibility to primary care has generated medical deserts. Identifying them is key to target the geographic areas where action is needed. An extensive definition of primary care has been promoted by the World Health Organization: a first level of contact with the health system, which involves the co-presence of different categories of health professionals alongside the general practitioner for the diagnosis and treatment of patients. Previous analyses have focused mainly on a single type of provider while this study proposes an integrated approach including various ones to define medical deserts in primary care.

**Method:**

Our empirical approach focuses on the first point of contact with the health system: general practitioners, proximity primary care providers (nurses, physiotherapists, pharmacies, laboratories, and radiologists), and emergency services. A multiple analysis approach was performed, to classify French municipalities using the information on the evolution and needs of health care accessibility, combining a principal component analysis and a hierarchical ascending classification.

**Results:**

Two clusters of medical deserts were identified with low accessibility to all healthcare professionals, socio-economic disadvantages, and a decrease in care supply. In other clusters, accessibility difficulties only concern a part of the health supply considered, which raises concern for the efficiency of primary care for optimal healthcare pathways. Even for clusters with better accessibility, issues were identified, such as a decrease and high needs of health care supply, revealing potential future difficulties.

**Conclusion:**

This work proposes a multi-professional and multi-dimensional approach to medical deserts based mainly on an extensive definition of primary care that shows the relevance of the co-presence of various healthcare professionals. The classification also makes it possible to identify areas with future problems of accessibility and its potential consequences. This framework could be easily applied to other countries according to their available data and their health systems’ specificities.

**Supplementary Information:**

The online version contains supplementary material available at 10.1186/s12942-024-00366-7.

## Background

To achieve the best health outcomes for the population, one of the main healthcare policy targets consists of guaranteeing that the population has equal access to healthcare services regardless of location [1]. However, all countries face geographical imbalances of human resources in the healthcare sector (HHRs) for primary and/or specialized healthcare [2, 3].

That leads to a local shortage of HHRs specifically in rural or socially deprived urban areas [4] defining medical deserts also named medically underserved areas (MUAs). The identification of such areas became a major public health policy purpose to target areas for action to improve accessibility to health care in under-supplied areas. It is also a challenge because ‘the greatest obstacle to the application of the concept of accessibility lies in the difficulty of translating it in the form of operational indicators’ [5]. This is exacerbated by the fact that the accessibility itself is complex to address due to its multidimensional nature (spatial, physical, temporal, financial, and social) [6–8]. But despite being a broader concept, spatial accessibility is the one that needs to be targeted first by policy reforms to identify MUAs because access to health care depends on the spatial accessibility of services which is a major determinant of health care utilization [9, 10].

From a policy-making perspective, place-based measures in which the level of accessibility is associated with a place or spatial unit of analysis are generally used to measure spatial accessibility. They are usually preferred to other measures like individual-centered or utility measures [11] because they inform governments and land-use planners about areas with accessibility deficits and allow for the assessment of socio-spatial inequalities [12, 13]. In that way, the place-based measure defines accessibility in terms of the physical separation between the location of services and key locations in daily life such as the place of residence or the place of work. Several studies dealing with health care accessibility are computed by population-to-provider ratios and distance to services for measuring either availability or proximity [14]. Some studies used more complex indicators like x-floating catchment area (xSFCA) combining availability and distance to better specification of health care needs, and supply and are used in France, for example, to define MUAs [15–18]. Nevertheless, it is important to note that these unidimensional indicators do not give an overall view of disparities in access to care but rather a view by profession. While spatial accessibility received great attention from researchers over several decades, previous analyses were mostly focused on single types of providers [19, 20].

Inter-sectoral capacity planning is less investigated while strengthening integration, multi-professional cooperation, skill mixing, and development of advanced roles for paramedics are considered major mechanisms to improve the quality of care and performance in the delivery of care [21]. For a synthetic view of geographic disparities, some authors explore combining access scores for different types of care defining univariate index like Gao and al. [10] for an integrated index of spatial accessibility bringing together professionals to care for pregnant women and Siegel and al [22] for a composite index across ambulatory and inpatient care based on the concept of regional deprivation measurement. But this type of indicator does not allow to identify the particular domain leading to relatively poor accessibility. Other authors built spatial classification measuring both physical, social environment, and spatial accessibility for a global understanding of the spatial dimension of health inequalities [23, 24]. Nevertheless, they do not focus on accessibility to health care or add contextual factors for a more global view of medical deserts.

This paper aims to demonstrate the added value of a new comprehensive classification approach to address the multi-professional and multi-dimension of health care accessibility to deal with a medical desert definition describing the spatial main structure and the specificity of medical desert in France. Considering this challenge, we propose a spatial classification of municipalities to highlight combined effects on the level, evolution, and needs of health care accessibility. This study is part of a European Union-funded program named OASES (prOmoting evidence-bASed rEformS on medical deserts). Our work package in this program aims to share and exchange methods, tools, and practices on accessibility to care measures.

## Rational and scope of the study

WHO defines primary care as a process that supports first-contact, accessible, continued, comprehensive and coordinated patient-focused care [25]. So, functions are emphasized rather than necessarily a list of healthcare professionals. Thus, primary care has various forms depending on the countries and the roles of each healthcare workers [26]. For example, in Germany the primary care system is mainly based on general practitioners working in single practice whereas in Spain primary care is provided by multi-professional teams including a wider range of healthcare professionals (GPs, pediatricians, nurses, midwives, physiotherapists, and even social workers and administrative staff workers). Moreover, according to WHO [3, 25] health systems built on primary healthcare lead to better health outcome better cost-efficiency and are essential to achieve universal health coverage for populations.

French primary care policy has historically been built mainly on a liberal care policy provided by GPs and other specialists, with no planned care pathways. Since 2004, it has been explicitly organized around a voluntary but incentivized registration to a physician gatekeeper (“médecin traitant”), most of the time a general practitioner, and incentivized referral for patients to access on the other level of care (care pathway) which firstly introduces an explicit principle of hierarchy in the access to different level of care. In this organization, general practitioners are responsible for (i) responding to the various needs of primary care without accommodation, (ii) the patient’s entry into the healthcare system (excluding emergencies and direct access to certain medical specialties), (iii) referral to other medical or paramedical health professionals when necessary and the coordination of care pathways [26].

In line with what is mentioned above and according to their role, we propose three other groups of healthcare providers. We qualified nurses, physiotherapists, and pharmacies as “closest proximity providers” and medical laboratories and radiology practices as “intermediate proximity providers” to underline the required complementarity of these professions for the diagnosis and treatment of patients alongside the GPs which promotes a better patient-centered integrated approach of care [27, 28]. It also refers more broadly to their role in the healthcare system with regards to ambulatory shift (return to home and coordination process linked to a growing and changing demand for care due to the increased prevalence of chronic disease and multimorbidity). Beyond primary care, the proposal is to include emergency care which aims to get an immediate response to a need for care—especially when it is a life-threatening emergency- and essential [16].

## Method

### Context and geography

We focused on France’s mainland and overseas departments and performed our classification at the municipality level (34,990 municipalities). The years of reference of the geographical data were 2019 or 2020, corresponding to the most recent data provided by the different data sources (Table 1). Municipality was an area of interest because it serves as a basic unit for many statistics, and it is the smallest geographic unit for data available in some data sources such as medico administrative ones. Primary care is linked to the concept of proximity: the Alma-ata declaration describes primary care as “bringing health care as close as possible to where people live and work” [29]. The challenge is to have a diagnosis of accessibility that is both precise in its construction assumptions and in its results.

**Table 1:** List of active variables before creating the scores

Dimension	Scores	Variables	Time	data source	Scale
Health care accessibility		LPA of GPs^a^	2019	DREES	Municipality
	Closest proximity providers	LPA of nurses^b^	2019	DREES	Municipality
		LPA of physiotherapists^b^	2019	DREES	Municipality
		Distance to the nearest pharmacy^c^	2020	INSEE	Municipality
	Intermediate proximity providers	Distance to the nearest laboratory^c^	2020	INSEE	Municipality
		Distance to the nearest radiologist^c^	2020	SNDS	Municipality
		Distance to the nearest emergency service^c^	2020	FINESS-SAE-Metric	Municipality
Dynamic of supply		GPSs’ LPA annual average rate of change	2015–2019	DREES	Municipality
		Share of GPs over 60 years old	2020	FNPS/CNAM	Health living territories
Needs of healthcare		Standardized mortality rate^d^	2013–2017	Inserm-CepiDc, Insee—Fnors exploitation	EPCI
		Standardized premature mortality rate^d^	2013–2017	Inserm-CepiDc, Insee—Fnors exploitation	EPCI
		Median income per household	2019	INSEE	Municipality

^a^In number of accessible consultations per year per capita

^b^In accessible FTEs per 100,000 inhabitants

^c^In minutes

^d^Per 100,000 inhabitants

### Multiple dimensions indicators

In line with literature and knowledge, the first dimension is health care accessibility. Then, other dimensions have been included: the dynamic of supply (with the temporal evolution of GPs supply) and the needs of health care. The dynamic of supply will reveal growing inequalities in access to care resulting in both the decline in the number of self-employed general practitioners, contrary to other primary healthcare professionals, and their activity, but also, the growth and ageing of the population [30]. The needs of health care dimension qualify areas whose populations have higher needs, a priori, i.e., whose socioeconomic characteristics and/or state of health are unfavorable.

#### Accessibility to primary healthcare professionals and emergency services

GPs, nurses, and physiotherapists localized potential accessibility (LPA) indicators for all French municipalities based on the xSFCA method were included [17, 19, 31, 32]. By definition, the LPA is a density of health professionals’ full time equivalent or consultations available per year, in relation to the population standardized by sex and age. This density transcends administrative boundaries by considering the supply of care and demand in the geographical unit under consideration, but also that of the surrounding geographical units. These indicators have been used as a basis for French public policy zoning by profession. Moreover, previous studies have used them to analyze accessibility to health care to deal with social health inequalities [22, 24]. Calculated in routine by the Department of Research, Studies, Evaluation, and Statistics (DREES) of the Ministry of Health, the value represents the number of health professionals per 100,000 inhabitants for nurses and physiotherapists and the number of consultations accessible per year and inhabitants for GPs.

Distances in minutes to the closest radiologist, laboratory, pharmacy, and emergencies, including emergency services and emergency rapid response unit, were calculated to estimate the accessibility to these healthcare professions. Access time was calculated by car using the road network considering several parameters such as topography or network configuration and operation. All the distances were provided by a distance matrix developed by IRDES.

#### Dynamic of supply

To assess the evolution of accessibility, two variables were calculated. The first was the evolution of accessibility to GPs over the last few years with an average annual evolution rate of the LPA between 2015 and 2019 with data provided by the DREES in routine. Then, the percentage of general practitioners over 60 years of age by health living territories (groups of municipalities according to the possibilities of access for a given population to the most frequent daily facilities and services which reflects the organization of usual mobility in this territory) was calculated to foresee the future evolution in the health territory likely to retire in the following years.

#### Needs of health care

The age-differentiated needs of the population are already considered in the calculation of the LPA. We wanted to add to this score the level of socio-sanitary disadvantage of the population. For this purpose, after sensitivity analysis, we retained the median income as a proxy of the socio-economic level of municipalities. As a proxy of higher needs of care, the standardized global and premature mortality rates per 100,000 inhabitants were calculated at the level of public establishments for cooperation between local authorities (EPCI) which are groups of municipalities whose purpose is to carry out joint development projects (EPCI). Data comes from the National Federation of Regional Health Observatories (FNORS).

#### Other dimensions

The classification was intended to focus on measuring accessibility to healthcare. Dimensions that do not directly measure accessibility, the supply, or the demand side but rather explain it or qualify the territories have therefore not been included in the construction of the clusters but have been retained as illustrative variables. So, the urbanization degree, the level of attractivity and the local organization of healthcare have been used to describe groups of municipalities (see Additional file 1). Socio-economics characteristics and the dynamic of closest proximity providers supply have also been used as illustrative variables for a question of balance between the dimensions.

### Multiple statistical analysis approach

**Fig. 1:**
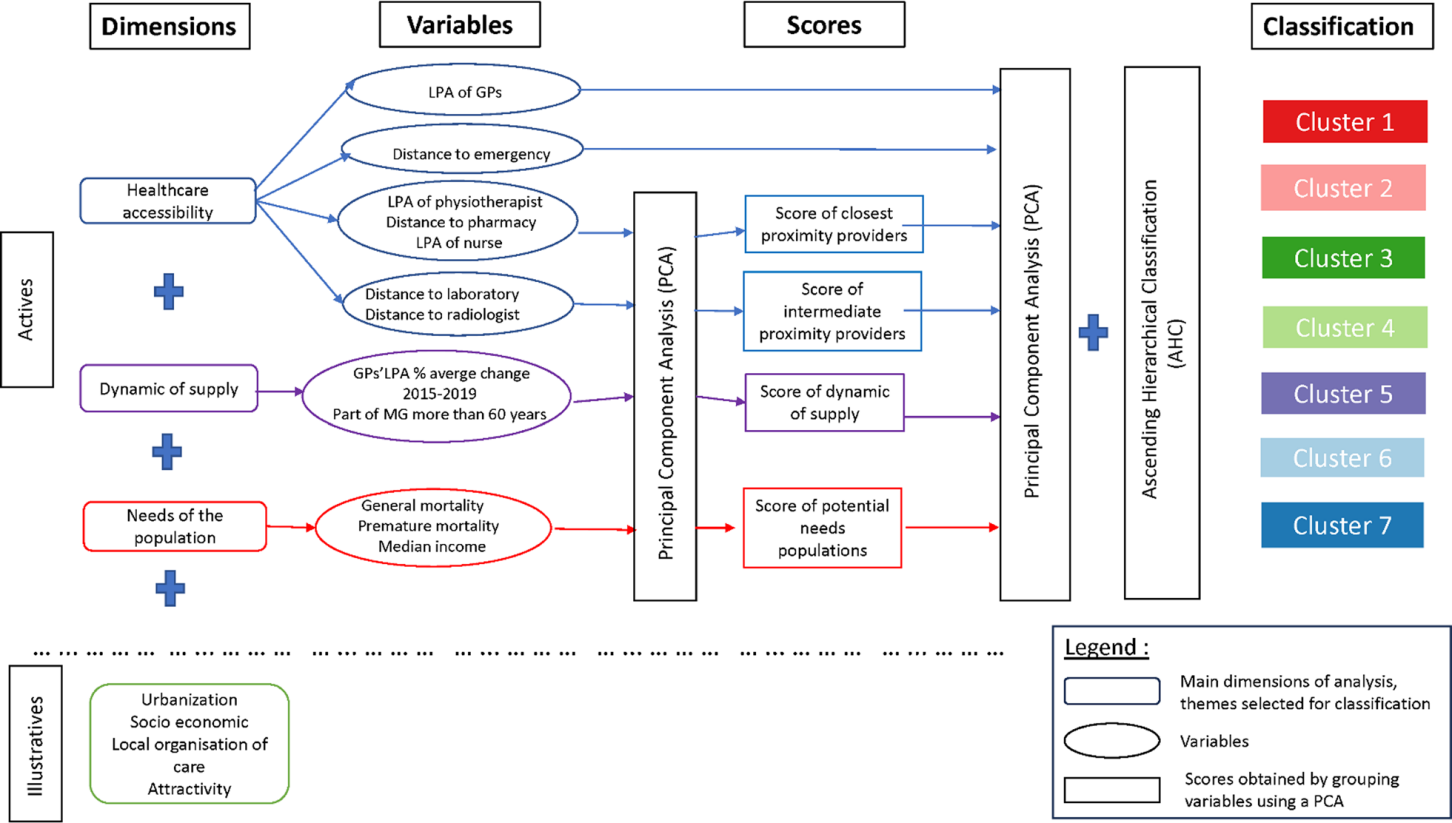
Processing steps to build a multidimensional classification

We propose a non-normative approach by using multivariate statistical analysis to classify French municipalities according to their level of access, evolution, and needs for health care accessibility. This approach uses a three-step method that consists in creating a score by dimension as a pre-processing step before performing a traditional combination of Principal Component Analysis (PCA) and the clustering method (Fig. 1). These approaches provided more statistical stability and robustness to the clustering process, thus minimizing the risk of territorial misclassification [33].

#### Building scores

Four scores were computed using a Principal Component Analysis (PCA) and taking the coordinates of the municipalities on the first axis as the score: closest proximity providers, intermediate proximity providers, dynamic of supply, and needs of health care (Fig. 1). Synthesizing groups of variables has already been used in previous work [23] and meets two main objectives:

1. To increase the proportion of information explained by the classification.
2. To give more weight to the dimensions we wanted to focus on. Creating a score for the dimensions dynamic of supply and needs of the population gives more weight to accessibility in the construction of the clusters, as these variables are more numerous.

Accessibility to general practitioners and emergencies are introduced directly into the analysis due to their distinction from the others considering that GPs are the cornerstone of health care accessibility, and emergencies concern the hospital setting.

#### Classifying French municipalities

**Fig. 2:**
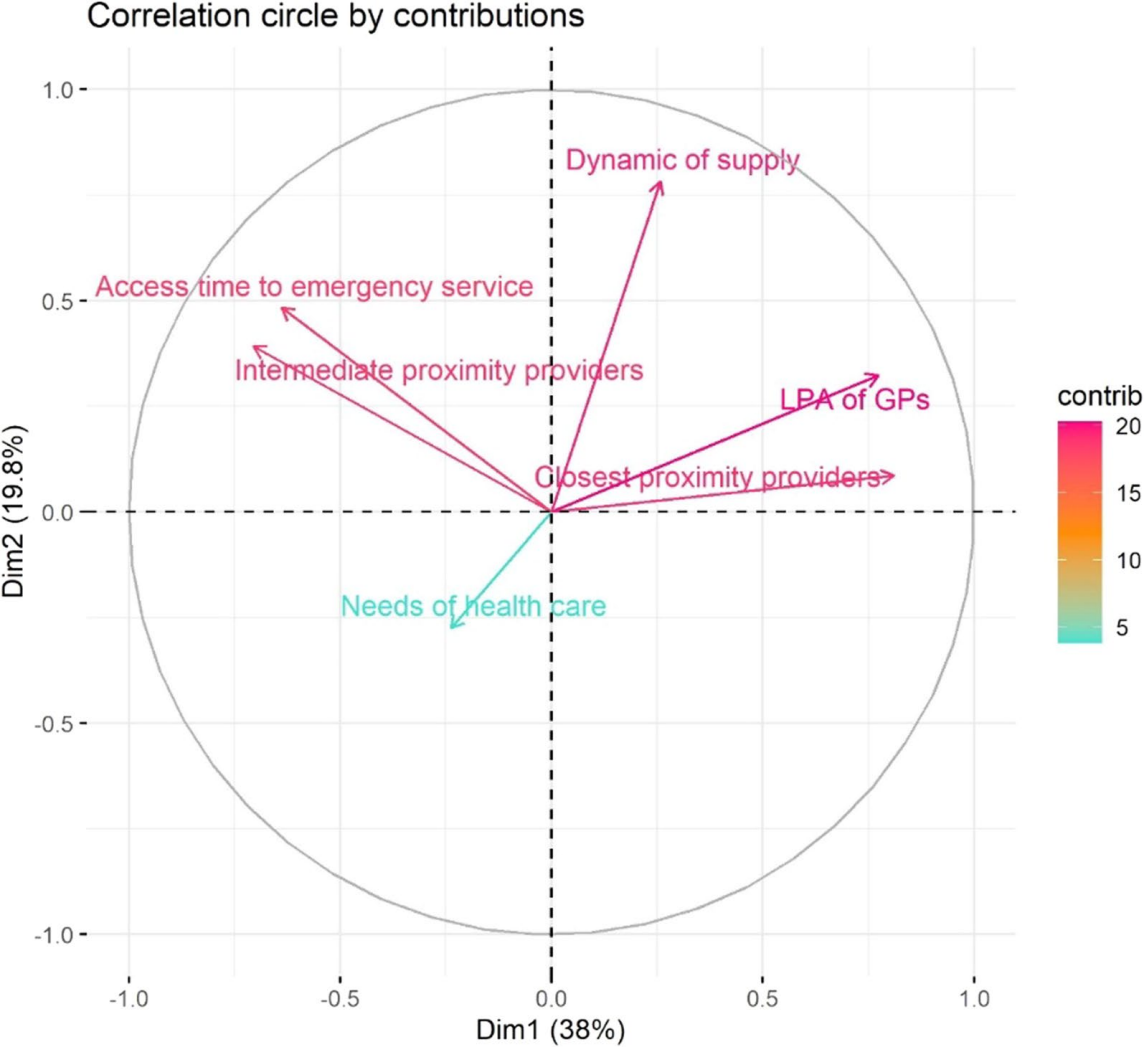
Results of the PCA

First, a global PCA was used to synthesize information including the two variables and the four scores to understand how they position in relation to each other and to see which variables most strongly discriminate municipalities in our analysis (Fig. 2). The percentage of variance explained by the first two components of the PCA is 57.8% (38% for the first axis and 19.8% for the second). In terms of hierarchy, we obtain all the variables of accessibility to health care contributing most strongly on the first axis, then the evolution of supply on axis 2, and the needs of the population on axis 3. The correlation circle reveals on the first axis an opposition between better-endowed and less-endowed areas with access times on one side and density on the other. This means that where the density of GPs and closest proximity providers is high, the travel time to emergencies and intermediate proximity providers is generally shorter. The second axis is dominated by the dynamic of supply, which with the LPA to the GPs, is slightly opposed to the needs of the population. In other words, the dynamic of supply would be better for areas already well endowed with GPs which would increase inequalities. On the contrary, accessibility tends to decrease in the most socio-economically disadvantaged areas.

In a second step, an ascending hierarchy classification (AHC) was performed to classify municipalities in categories according to all dimensions’ divers by nature (groups of professions, dynamic supply, and needs of healthcare). This method consists in grouping spatial units in relation to each other, according to their similarities (within a cluster) and dissimilarities (between clusters) based on their characteristics defined by a set of variables. Based on the analysis of the inertia gain graph and the dendrogram, a classification of 7 clusters gains the most information summarized while keeping a reasonable number of clusters. Then, other illustrative dimensions will be integrated to describe the 7 clusters. Statistical analyses were performed using R version 4.2.1 (2022-06-23).

## Results

### Presentation of municipalities’ clusters of medical deserts

**Fig. 3:**
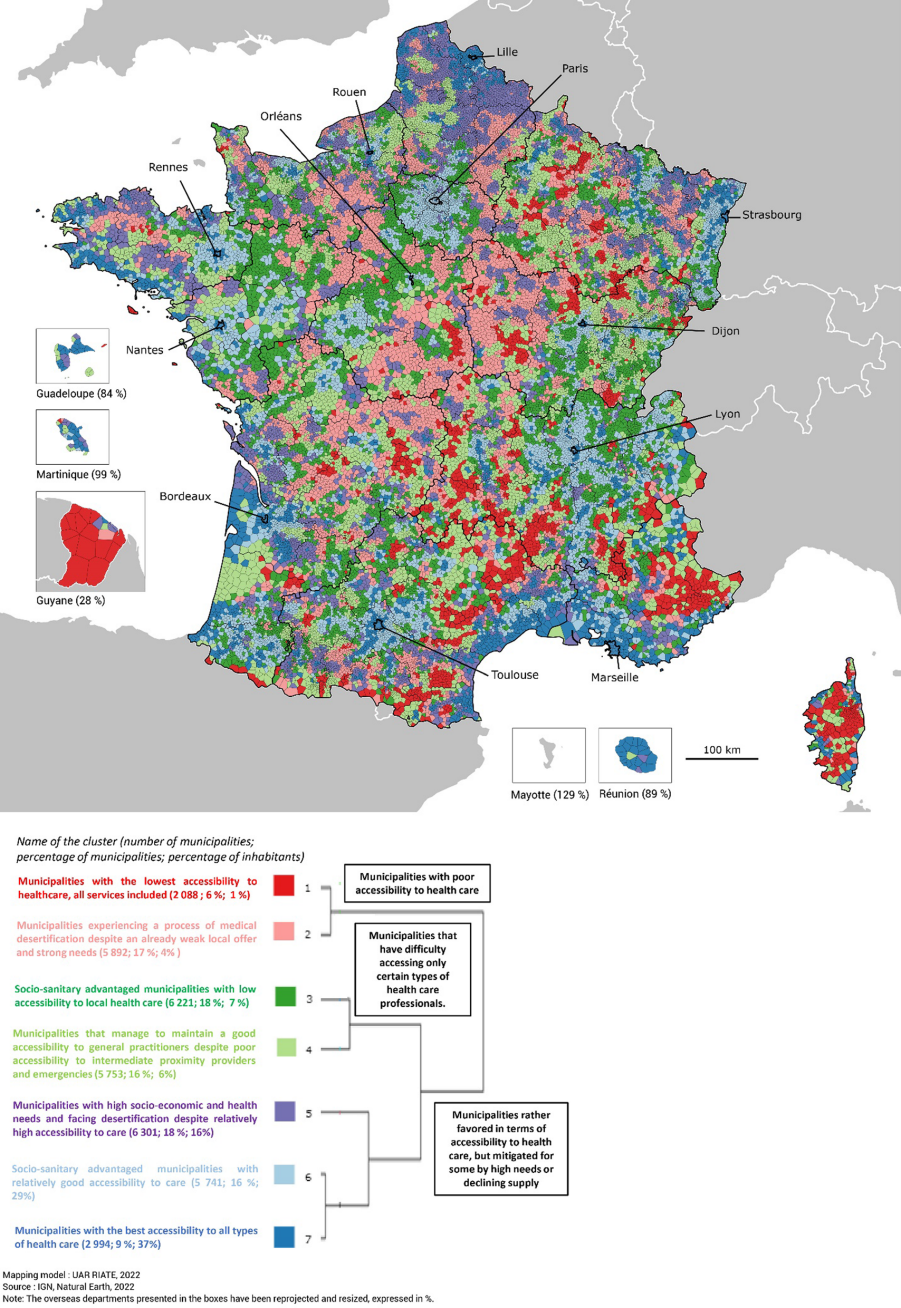
Geographic distribution of clusters in Metropolitan France and overseas departments

**Fig. 4:**
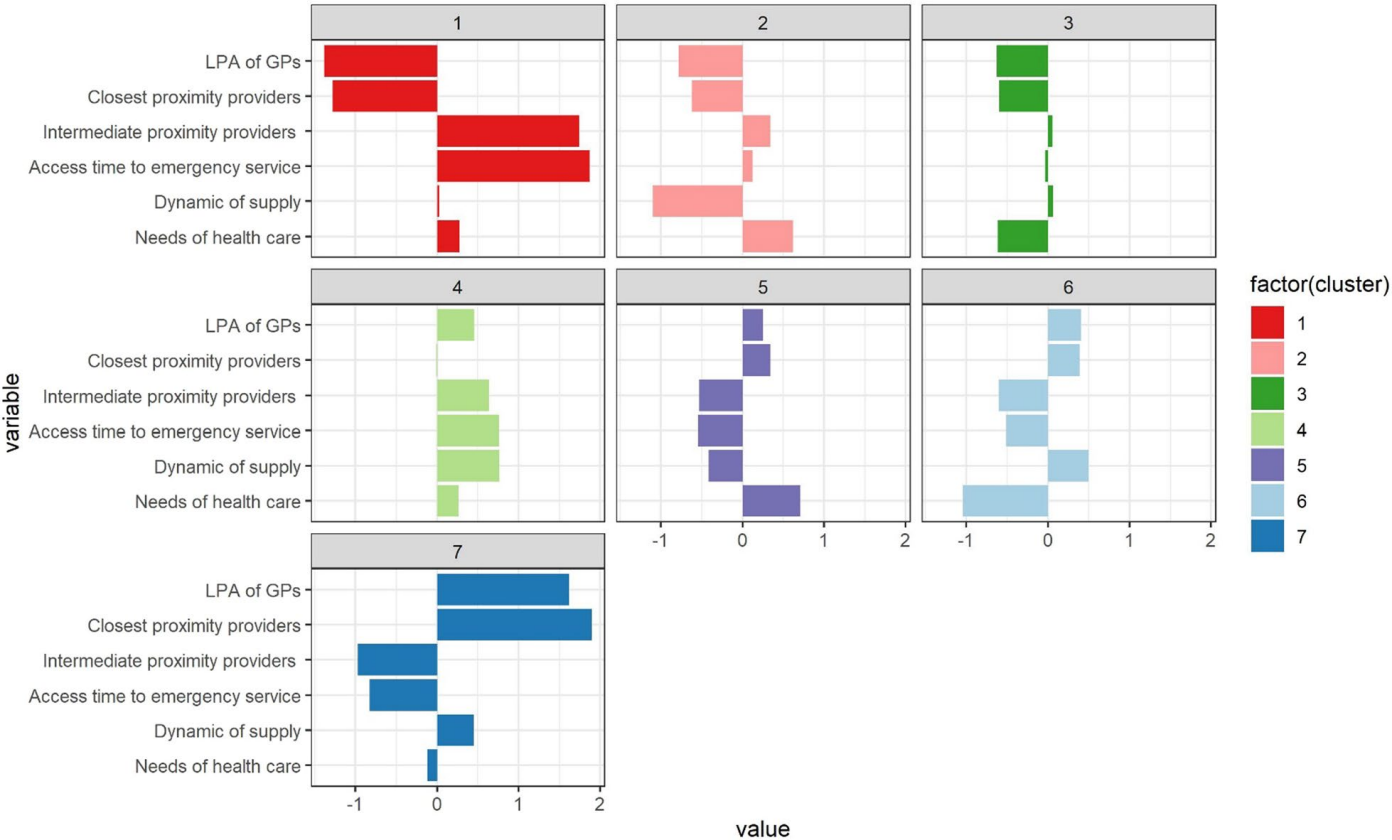
Standardized cluster profiles. *Each bar represents the distance from the average of each cluster to the overall average. This distance is expressed as the number of standard deviations of each variable.

Figure 3 presents the geographic distribution of clusters in Metropolitan France and overseas departments while Table 2 and Fig. 4 describe them in comparison to the national average of municipalities according to their level, evolution, and needs of primary health care accessibility. To go further, a table and graphs are provided in Additional files 2 and 3, giving the characteristics of the clusters according to illustrative variables.

**Table 2:** Description of the clusters according to active variables

Characteristics, mean (SD)	*Isolated rural municipalities with poor accessibility to all healthcare professionals and services*		*Rural or outlying suburban municipalities that have difficulty accessing only certain types of healthcare professionals*		*Urban centers rather favored in terms of accessibility to health care, mitigated for some by high needs or declining supply*			Global^a^
	Cluster 1	Cluster 2	Cluster 3	Cluster 4	Cluster 5	Cluster 6	Cluster 7	
Part of municipalities (%)	6	17	18	16	18	16	9	
Part of inhabitants (%)	1	4	7	6	16	29	37	
Health care accessibility								
LPA of GPs^b^	1.1 (1.0)	1.9 (0.7)	2.0 (0.6)	3.3 (1.0)	3.1 (0.6)	3.3 (0.7)	4.7 (1.0)	2.8 (1.2)
LPA of nurses^c^	61.7 (58.8)	81.7 (35.7)	79.0 (31.9)	114.4 (59.1)	137.6 (52.9)	121.9 (44.5)	218.7 (96.2)	113.8 (66.3)
LPA of physiotherapist^c^	25.0 (27.8)	39.5 (19.7)	45.8 (19.9)	63.9 (33.4)	73.9 (25.5)	87.5 (28.7)	145.9 (46.6)	66.9 (41.6)
Distance to the nearest pharmacy^c^	11.6 (6.4)	6.8 (3.6)	7.2 (3.8)	5.5 (3.7)	4.5 (3.5)	4.4 (3.9)	2.3 (3.3)	5.8 (4.4)
Distance to the nearest laboratory^d^	27.8 (12.7)	17.4 (6.1)	15.8 (5.5)	19.2 (8.7)	11.1 (5.4)	10.6 (5.6)	7.6 (6.2)	14.9 (8.5)
Distance to the nearest radiologist^d^	36.8 (16.3)	23.4 (7.9)	20.1 (6.7)	26.7 (9.8)	15.0 (7.5)	14.2 (6.9)	11.0 (7.8)	20.1 (10.8)
Distance to the nearest emergency service^d^	49.4 (17.2)	27.1 (8.7)	24.9 (7.8)	35.1 (10.6)	18.4 (8.3)	18.7 (7.5)	14.7 (9.4)	25.4 (12.8)
Dynamic of supply								
Gps’ LPA annual average rate of change	− 1.9 (4.9)	− 5.9 (4.0)	− 2.6 (3.4)	0.3 (4.4)	− 3.5 (2.9)	− 1.0 (2.5)	− 1.0 (2.4)	− 2.37 (4.1)
Part of GPs over 60 years old	35.0 (18.0)	48.3 (19.7)	30.7 (14.5)	23.8 (15.7)	40.2 (15.4)	25.6 (12.9)	27.1 (12.6)	33.3 (17.9)
Needs of healthcare								
Standardized general mortality rate^e^	978.4 (90.7)	1027.5 (89.7)	918.1 (67.4)	993.8 (86.5)	1035.1 (96.4)	892.0 (67.6)	952.1 (96.7)	972.2 (100)
Standardized premature mortality rate^e^	206.0 (34.0)	221.9 (29.0)	182.5 (26.5)	207.7 (30.3)	229.6 (31.5)	171.4 (25.6)	204.0 (36.2)	203.19 (36.3)
Median income per household	19,759 (2357)	20,585 (1936)	22,676 (3141)	20,733 (2015)	20,986 (2010)	24,230 (3595)	22,119 (2814)	21,733 (2970)

^a^Average based on municipalities. It is a territorial average and not a population average

^b^In number of accessible consultations per year per capita

^c^In accessible FTEs per 100,000 inhabitants

^d^In minutes

^e^Per 100,000 inhabitants

#### Isolated rural municipalities with poor accessibility to all healthcare professionals and services (clusters 1 and 2)

The first two clusters in red and pink are composed of rural areas with very sparse population that are rather isolated or situated in the peri-urban area of small urban centers (see Additional files 2 and 3). They are characterized by an accumulation of low accessibility to all health care services and workers and by a significant level of socio-sanitary disadvantage. The municipalities of cluster 1 are more difficult to access and are often located in mountainous areas (southern Alps, Corsica), on islands, or in certain French overseas departments (Guyana). It concerns a few municipalities (6%) and people (1%). It encapsulates outliers with the lowest accessibility to all health services while the needs are higher than the average accentuated by a large proportion of elderly people. Cluster 2 can be found in the northern half of France. It is distinguished from cluster 1 by its desertification process. In addition to lower accessibility, especially for the local supply, and a higher level of need underlined by the high unemployment rate and share of blue-collar workers, this is the cluster with the most significant loss of accessibility to general practitioners. This is accentuated by a lower increase in accessibility to nurses and physiotherapists compared to the average.

#### Rural or outlying suburban municipalities that have difficulty accessing only certain types of healthcare professionals raising concern for the efficiency of the primary care system (clusters 3 and 4)

The next two clusters in dark and light green are mainly located in rural or remote suburban areas. But contrary to the previous clusters, accessibility difficulties only concern a part of the health supply considered, which raises concern for the efficiency of primary care for optimal healthcare pathways. Cluster 3 is peri-urban. Its municipalities often belong to the outskirts of medium-sized or large cities like Rennes, Lyon, or Dijon, privileged from the socio-sanitary point of view with a significant percentage of higher education graduates. It is rather average in terms of accessibility to emergencies or intermediate proximity providers thanks to its relative proximity to urban centers, while its accessibility to local services such as general practitioners is poorer. It is an issue because this cluster is characterized by a growth of its population which may indicate a future increase in needs. The municipalities belonging to cluster 4 are generally more isolated or in the peri-urban area of smaller centers than the municipalities of cluster 3. They are less privileged municipalities from a social and health point of view with a high proportion of elderly people. At the regional level, they are often in the southwest of the country but also the northeast. For this cluster, the opposite phenomenon of cluster 3 occurs. Accessibility is highest for general practitioners but rather poor for radiologists and laboratories, and emergencies. Another point that differentiates the two clusters concerns the dynamic of the supply. Indeed, cluster 4 has seen its accessibility to general practitioners maintained or even increased. This is the cluster that is experiencing the most favorable dynamics even though it is a rather rural cluster. But it should be noted that this dynamic only concerns GPs, the evolution of closest proximity providers being rather average.

#### Urban centers rather favored in terms of accessibility to health care, mitigated for some by high needs or declining supply (clusters 5, 6, and 7)

The three last clusters in purple, light, and dark blue are made up of centers located at different levels of the urban hierarchy. These clusters have the particularity of being well endowed with all types of health care. Thus cluster 5, mainly composed of small and medium-sized centers in the North of Metropolitan France, stands out from the other two clusters because it has the highest level of socio-sanitary disadvantages of all the clusters combined. The illustrative variables highlight it with a high unemployment rate and share of blue-collar workers. This cluster is also experiencing a process of desertification. This is the cluster, after cluster 2, with the most important decrease in GPs and the lower evolution of accessibility to physiotherapists. Clusters 6 and 7 share the same good level of accessibility but do not face the same issues of the evolution of GPs and needs. The peri-urban municipalities of major centers constituting cluster 6, are distinguished by their strong level of socio-sanitary advantages with a considerable proportion of higher education graduates. The supply dynamic is rather favorable which is important because these municipalities may face a rise in needs in the coming years as they experience an increase in their general population particularly for children and the elderly requiring more health care. Finally, municipalities of cluster 7 are in urban centers at all levels of the urban hierarchy but also in overseas departments like Reunion or metropolitan islands like Ouessant island. It is the counterpart of cluster 1, but this time in terms of better access to care. It brings together the extreme values and outliers with the greatest accessibility to care. The dynamic of supply is among the most favorable for GPs as well as for closest proximity providers. However, this cluster faces some challenges. It is characterized by a strong social heterogeneity (high share of higher education graduates, unemployment rate, and proportion of single-parent families) and by a growing population that could lead to an increase in demand.

### Main spatial structures highlighted by the distribution of the clusters

The distribution of the clusters draws mainly two types of spatial structures: an urban–rural gradient and regional north/south contrasts for Metropolitan France.

First, this classification shows a classic urban/rural gradient for emergency services and intermediate diagnostic partner with better accessibility to the urban center (clusters 7 and 5), then good accessibility in the inner ring (cluster 6), progressively moving into more distant suburbs with average accessibility (cluster 3) and ultimately lower accessibility in the rural margins (clusters 4, 1 and 2). However, this gradient is not the same for closest proximity services like GPs, nurses, physiotherapists, and pharmacies. Beyond the classic urban/rural opposition, it is the importance of small centralities in providing accessibility to proximity services that is emphasized. Cluster 4, although situated in isolated rural areas, has an as high level of accessibility to general practitioners as clusters 5 and 6, and even better than cluster 3, which is closer to large urban centers. It is because cluster 4 is structured in small towns that play the role of local centralities and allow them to be well served by local services. This illustrates the role of small towns in providing the supply of care in rural areas. Accessibility no longer depends on distance from large cities but on distance from local service and equipment centers (see Additional file 3).

The classification also underlines a North/South pattern for Metropolitan France in terms of socio-sanitary disadvantages and dynamic of supply which reveals a broader opposition between more and less attractive areas. Clusters 2 and 5, the most disadvantaged in terms of socio-sanitary conditions and with the largest loss of accessibility to GPs, are both located in the north of France which appears to be less attractive, with a high proportion of vacant dwellings, a drop in the number of inhabitants and a low migratory balance. On the contrary, cluster 7 which is on the Atlantic and Mediterranean coasts and cluster 6 which is on attractive suburban areas manage to maintain a good supply of GPs and have the opposite characteristics. Some patterns concerning Primary care team practices (PCTs) can also be evidenced. PCTs are primary care structures, grouping at least two general practitioners and one paramedic, based on a collective and coordinated mode of practice. Cluster 2, has a rather low share of municipalities equipped with PCTs within their health living territories for rural areas (71%), whereas cluster 4, which is more able to attract GPs, is rather well equipped (78%).

## Discussion

In a French context of medical desertification that is likely to last for several more years, the objective of this work was to characterize municipalities according to their accessibility to GPs and closest proximity providers, the evolution of the medical supply, and the characteristics of the populations. From a non-normative approach, this work brings lessons for the French case but also for countries that seek to qualify “medical deserts”. We have taken into account several primary care healthcare workers to define, from multivariate statistical analyses, different clusters of territories according to their accessibility to them. Moreover, to complete this description, contextual factors well known to influence positively or negatively their accessibility are included. Our results show 7 clusters of municipalities. Two of them stand out as “medical deserts” compared to others. These clusters accumulate unfavorable situations in all domains such as low accessibility to multiple professions, high needs, and low dynamic of supply, and the inverse clusters which accumulate favorable ones. In addition, the typologies highlight areas where the level of accessibility of some workers is more mixed raising concern for the efficiency of primary care, to which must be added the non-negligible influences of the level of needs and the evolution of the supply of care revealing potential difficulties.

Approaches defining access to care across territories are more often based on access to a single healthcare professional [34] and also in France [17, 20]. Research in Germany has analyzed access to multiple healthcare professionals with a composite index [10]. Our study completes these field by proposing a method for defining territorial disparities in accessibility to primary care.

As an empirical and non-normative approach, our method implies a definition of medical desert by characterizing situations comparatively as better or worse by statistical approach rather than a normative one which implies a definition of thresholds that need to be made explicit upstream of the public debate and then within it. Using the clustering process, for its part, is relevant to distinguish areas with specific combinations of geographical features. The methodology is of interest to all countries seeking to characterize medical deserts with multiple health professionals and will have to be adapted to the healthcare system of each country.

The choice to use scores summarizing different dimensions concerning GPs, closest proximity providers and the population makes it possible to identify several configurations. Through the creation of groups, the quality of the classification (by the part of information explained: 42.9% of the information was summarized by the first two axes without scores against 57.8% with scores) improved the reading of the results. Moreover, it allows highlighting professions according to their importance in the healthcare system giving them more weight. Nonetheless, the method requires a few choices about how to evaluate accessibility indicators, which domains are more or less important, and which services should be selected to form a domain that designs the study.

Then, the municipality scale illustrates the heterogeneity of rural areas, their dynamics, and their polarities. On the other hand, for large urban areas, and especially the region Ile-de-France, the scale does not provide any added value: most of the municipalities appear with better accessibility and are favored over the average, while inequalities in access to GPs are very high. This limitation can be circumvented by using finer scales of study for the largest agglomerations.

Our research confirms results previously demonstrated in others studied based on access to GPs and/or closest proximity providers [16, 20, 24]. This is the case for clusters 1 and 2 of rural municipalities with lower accessibility to care and less attractiveness mainly located inland or in the northern half of France. The better medically served areas such as the coasts or the big cities stand out as well here in cluster 7. Previous study suggests that better health care accessibility in urban areas in Germany persists when taking a holistic view [10]. Moreover, our results revealed that Cluster 4, although situated in isolated rural areas, has a rather high level of accessibility to GPs. This finding of high density in isolated rural areas has already been underlined in other studies on GPs [10, 19] but also, for other local services in various fields such as commerce, health and social action, education, sports, and leisure and tourism [34, 35]. This is explained by the role of small centralities in the provision of local health services in those areas.

As mentioned above, in a context where primary care needs to be structured around several healthcare professionals, it is important to have a multidisciplinary diagnosis. Our results provide specific new insights for France due to a multi-professions and multi-domains approach. Some clusters have similar levels of accessibility to GPs but differ in their accessibility to other providers which raises questions in terms of the care pathway (clusters 4 and 5). Other dimensions make it possible to nuance the observation by distinguishing clusters that have the same level of accessibility but not the same needs and dynamic of the supply and to highlight some clusters with probable difficulties to come (cluster 5). The description of the clusters using additional variables, such as the urban hierarchy or the level of equipment (see Additional file 3) reveals different levels of polarities in rural areas.

Our study proposes a method for describing potential interactions between professionals on a fine-scale territory. These interactions could reduce territorial disparities in accessibility to primary care and improve the quality of the healthcare system. Indeed, health systems based on primary health care deliver better health outcomes, are more cost-effective and make a key contribution to achieving universal health coverage for the population [3, 25]. They are essential in terms of prevention and early management of health problems which help to reduce hospital admissions and cut costs for the healthcare system [36].

Knowing that, these different municipal “configurations” can be inspiring for public authorities at both national and local levels to (i) question how areas that are under-dense in terms of health professionals are defined and (ii) complete or adapt existing measures to combat against “medical deserts”. For example, it is difficult to rely on other health professionals when there is a shortage in the area. Other systems must then be designed to ensure continuity of care (e.g. mobile services, delocalized consultations) on a coherent territorial scale. Conversely, in certain medically underserved areas with a good level of other healthcare professionals, multi-professional and coordinated healthcare organizations should continue to be encouraged, knowing the efficiency gains generated for GPs. All in all, these different municipal configurations implicitly illustrate the difficulty of defining under-resourced areas when several health professionals are taken into account. This difficulty, which we do not have the ambition to answer here, could be discussed during the definition of the next medically underserved areas.

Our study was carried out at the municipal level, but other scales could be relevant to analyze and describe the medical deserts. Groups of municipalities (EPCI) or the “territoire de vie sante” seem relevant scales in France for comparing diagnosis and decision in terms of medical desert measures to assess the level of similarities and differences. EPCIs are increasingly involved, locally, in improving access to care for their inhabitants [37] and the “territoire de vie sante” is the zoning used by French regional agencies to identify the priority areas for the location of GPs. To address this question, we plan to extend this work to a multiscale approach and apply this methodology at these two scales.

## Conclusion

As accessibility to care is a major issue for health policies due to geographical imbalances of health resources, this paper aims to demonstrate the value of a new comprehensive classification approach on accessibility to health care in a nationwide geographical perspective designed at the municipality scale. This multi-professional classification of municipalities based mainly on an extensive definition of primary care is a useful proposition to widen the scope considered to other professionals than GPs to underline the required complementarity of them for the diagnosis and treatment of patients alongside the GPs. In that way, it promotes a better patient-centered integrated approach to care that could be considered a useful tool to inform public policies and identify areas that warrant specific intervention. It could also easily be applied to other countries depending on the data available and the specificities of their health systems.

### Supplementary Information

**Additional file 1.** List of illustrative variables. List of illustrative variables with their sources.

**Additional file 2.** Description of the clusters by illustrative variables. A table containing the mean and standard deviation of illustrative variables by cluster.

**Additional file 3.** Description of the clusters according to urbanization degree. Diagrams showing the composition of clusters by urban area and level of centrality.

## Data Availability

All data of the geographical scores and the classification presented in this study are available from the corresponding author on reasonable request except for mortality data supplied by the FNORS, which are not available for distribution.

## Abbreviations

ANCT: Agence Nationale de la Cohésion des territoires (France’s Agency for Territorial Cohesion)
ARCEP: Autorité de Régulation des Communications Electroniques et des Postes (the French telecommunications and postal regulatory body)
CESAER: Centre d'Economie et de Sociologie Appliquées à l'Agriculture et aux Espaces Ruraux (Centre for Economics and Sociology Applied to Agriculture and Rural Areas)
CNAM: Caisse Nationale d’Assurance Maladie (National Health Insurance Fund)
DREES: Direction de la Recherche, des Études, de l'Évaluation et des Statistiques (Directorate for Research, Studies, Assessment and Statistics, French Ministry of Health)
FINESS: Fichier National des Etablissements Sanitaires et Sociaux (National File on Health and Social Institutions)
FNORS: Fédération Nationale des Observatoires Régionaux de la Santé (National Federation of Regional Health Observatories)
FNPS: Fichier National des Professionnels de Santé (National register of health professionals)
GP: General Practitioners
INRAE: Institut National de la Recherche Agronomique (National Institute for Agronomic Research)
INSEE: L’Institut National de la Statistique et des Etudes Economiques (National Institute of Statistics and Economics Studies)
INSERM: Institut National de la Santé et de la Recherche Médicale (National Institute for Health and Medical Research)
IRDES: Institut de Recherche et de Documentation en Economie de la Santé (institute for Research and Information in Health Economics)
MUAs: Medically underserved areas
SAE: Statistique Annuelle des Etablissements (Annual Statistics of healthcare facilities)
SNDS: Système National des Données de Santé (National health data system)
