# Supplementary material for: A French classification to describe medical deserts: a multi-professional approach based on the first contact with the healthcare system
Source: Int J Health Geogr. 2024 Feb 28;23:5. doi: 10.1186/s12942-024-00366-7 (PMC10900694; doi:10.1186/s12942-024-00366-7)
Supplement: Supplementary file 1 — Additional file 1. List of illustrative variables. List of illustrative variables with their sources. [file 12942_2024_366_MOESM1_ESM.pdf]

Dimension	Variables	Time	Data Source
Dynamic of the supply	Nurses' LPA annual average rate of change (in %)	**2016-2019**	**DREES**
	Physiotherapists' LPA annual average rate of change (in %)	**2016-2019**	**DREES**
Socio-economic characteristics of the population	Unemployment rate (in %)	**2018**	**INSEE**
	Percentage of blue-collar workers (in %)	**2018**	**INSEE**
	Percentage of employees (in %)	**2018**	**INSEE**
	Percentage of higher education graduates (in %)	**2018**	**INSEE**
	Percentage of single parent families (in %)	**2018**	**INSEE**
Demographic structure	Part of the population under 5 years old	**2018**	**INSEE**
	Part of the population over 65 years old (in %)	**2018**	**INSEE**
	Average annual growth rate of population (in %)	**2013-2018**	**INSEE**
	Average annual growth rate of people over 65 (in %)	**2013-2018**	**INSEE**
	Average annual growth rate of children under 5 years old (in %)	**2013-2018**	**INSEE**
Attractivity of the territory	Percentage of vacant housing (in %)	**2018**	**INSEE**
	Percentage of secondary residence (in %)	**2018**	**INSEE**
	Percentage of premises eligible for fiber (in %)	**2021**	**ARCEP**
	Percentage of commuters (in %)	**2018**	**INSEE**
	Annual net migration (in %)	**2013-2018**	**INSEE**
Urbanization degree	Density (in inhabitants per km2)	**2021**	**INSEE**
	City catchment area zoning	**2021**	**INSEE**
	Level of equipment and service centers	**2021**	**INRAE-CESAER- ANCT**
Local organization of healthcare	Presence of a multi-professional health center in the health living territory	**2020**	**FINESS**

* All variables are at the municipality level
