# Supplementary material for: A French classification to describe medical deserts: a multi-professional approach based on the first contact with the healthcare system
Source: Int J Health Geogr. 2024 Feb 28;23:5. doi: 10.1186/s12942-024-00366-7 (PMC10900694; doi:10.1186/s12942-024-00366-7)
Supplement: Supplementary file 2 — Additional file 2. Description of the clusters by illustrative variables. A table containing the mean and standard deviation of illustrative variables by cluster. [file 12942_2024_366_MOESM2_ESM.pdf]

	Isolated rural municipalities with poor accessibility to all health care professionals and services.		*Rural or outlying suburban municipalities that have difficulty accessing only certain types of health care professionals*		*Poles rather favored in terms of accessibility to primary care, but which may experience other difficulties.*			
**Indicators**	**Cluster 1**	**Cluster 2**	**Cluster 3**	**Cluster 4**	**Cluster 5**	**Cluster 6**	**Cluster 7**	**Global***
**Dynamic of supply**								
Nurses' LPA annual average rate of change (%)	2.1 (4.5)	2.2 (4.1)	2.3 (3.7)	2.5 (4.0)	2.9 (3.0)	2.5 (3.0)	2.9 (2.7)	2.5 (3.6)
Physiotherapists' LPA annual average rate of change (%)	2.6 (7.5)	1.7 (6.4)	2.0 (5.5)	2.4 (6.2)	2.0 (4.6)	2.7 (3.9)	3.6 (3.3)	2.3 (5.4)
**Socio-economic characteristics of the population**								
Unemployment rate (%)	12.6 (8.8)	11.4 (5.0)	9.0 (4.0)	10.6 (5.0)	11.8 (4.9)	8.6 (3.2)	11.9 (5.3)	10.6 (5.1)
Percentage of blue-collar workers (%)	23.0 (17.8)	28.3 (14.3)	25.3 (12.6)	27.7 (14.0)	28.3 (11.2)	21.2 (9.6)	21.9 (8.1)	25.6 (12.8)
Percentage of employees (%)	25.4 (17.7)	26.8 (12.7)	25.8 (10.1)	26.9 (12.0)	28.3 (9.4)	25.8 (7.4)	28.8 (6.4)	26.9 (10.8)
Percentage of higher education graduates (%)	22.0 (9.2)	20.8 (6.7)	25.9 (8.0)	21.6 (7.1)	21.9 (6.4)	31.3 (9.9)	28.5 (8.7)	24.5 (8.8)
Percentage of single parent families (%)	12.6 (15.4)	11.0 (10.3)	10.1 (8.3)	11.3 (10.0)	12.0 (7.7)	11.0 (6.1)	14.2 (7.0)	11.4 (9.1)
**Demographic structure**								
Part of the population under 5 years old (%)	3.8 (2.6)	4.8 (2.1)	5.4 (1.9)	4.8 (2.1)	5.1 (1.7)	5.5 (1.5)	5.3 (1.4)	5.1 (1.9)
Part of the population over 65 years old (%)	30.2 (10.0)	23.9 (7.9)	20.6 (6.8)	24.2 (8.2)	21.9 (6.5)	19.3 (5.6)	21.1 (5.9)	22.4 (7.6)
Average annual growth rate of population (%)	-0.2 (1.5)	-0.2 (1.3)	0.2 (1.3)	-0.1 (1.3)	0.0 (1.2)	0.5 (1.2)	0.5 (1.1)	0.1 (1.3)
Average annual growth rate of children under 5 years old (%)	-2.6 (6.2)	-3.0 (6.3)	-2.4 (6.0)	-2.7 (6.1)	-2.3 (5.5)	-1.3 (5.1)	-1.4 (4.4)	-2.3 (5.8)
Average annual growth rate of people over 65 (%)	2.2 (3.1)	2.5 (2.8)	3.2 (2.8)	2.5 (2.7)	3.0 (2.5)	3.8 (2.4)	3.5 (2.1)	3.0 (2.7)
**Attractivity of the territory**								
Percentage of vacant housing (%)	9.3 (6.3)	10.2 (5.4)	8.3 (4.3)	9.6 (5.1)	8.8 (4.2)	7.0 (3.3)	7.4 (3.6)	8.7 (4.7)
Percentage of secondary residence (%)	37.5 (20.2)	16.5 (13.4)	11.9 (12.1)	17.4 (17.0)	8.6 (10.7)	6.1 (9.1)	8.0 (13.9)	13.2 (15.3)
Percentage of premises eligible for fiber (%)	16.6 (34.5)	30.7 (43)	33.3 (42.2)	28.7 (41.6)	46.1 (44.8)	48.4 (42.1)	57.4 (39.3)	38.0 (43.4)

Percentage of commuters (%)	63.6 (18.3)	77.0 (12.3)	80.9 (10.5)	74.5 (14.6)	79.8 (11.8)	82.5 (9.5)	75.7 (16.5)	77.8 (13.6)
Annual net migration (%)	0.2 (2.5)	-0.1 (1.7)	0.0 (1.5)	0.0 (1.7)	0.0 (1.4)	0.3 (1.3)	0.4 (1.3)	0.0 (1.6)
**Urbanization degree**								
Density of population (inhabitants by km2)	12.2	34.8	60.2	43.9	147.5	493.9	559.8	180
Part of isolated municipalities outside the influence of urban centers (%)	63.3	37.1	20.1	42.4	16.4	8.5	7.1	25.5
Part of municipalities belonging to the ring of a major urban center (%)	8.9	13.6	33.4	26.1	14.6	51.4	39.2	27.5
Part of municipalities belonging to a major urban center (%)	0.0	0.0	0.2	0.0	1.0	6.6	10.9	2.2
**Local organization of healthcare**								
Part of municipalities belonging to a health living territory with an PCTs (%)	76.4	71.3	69.7	78.1	71.1	58.3	57.5	69.0

*Average based on municipalities. It is a territorial average and not a population average.
