# Supplementary material for: A French classification to describe medical deserts: a multi-professional approach based on the first contact with the healthcare system
Source: Int J Health Geogr. 2024 Feb 28;23:5. doi: 10.1186/s12942-024-00366-7 (PMC10900694; doi:10.1186/s12942-024-00366-7)
Supplement: Supplementary file 3 — Additional file 3. Description of the clusters according to urbanization degree. Diagrams showing the composition of clusters by urban area and level of centrality. [file 12942_2024_366_MOESM3_ESM.pdf]

Urban area zoning of municipalities by cluster

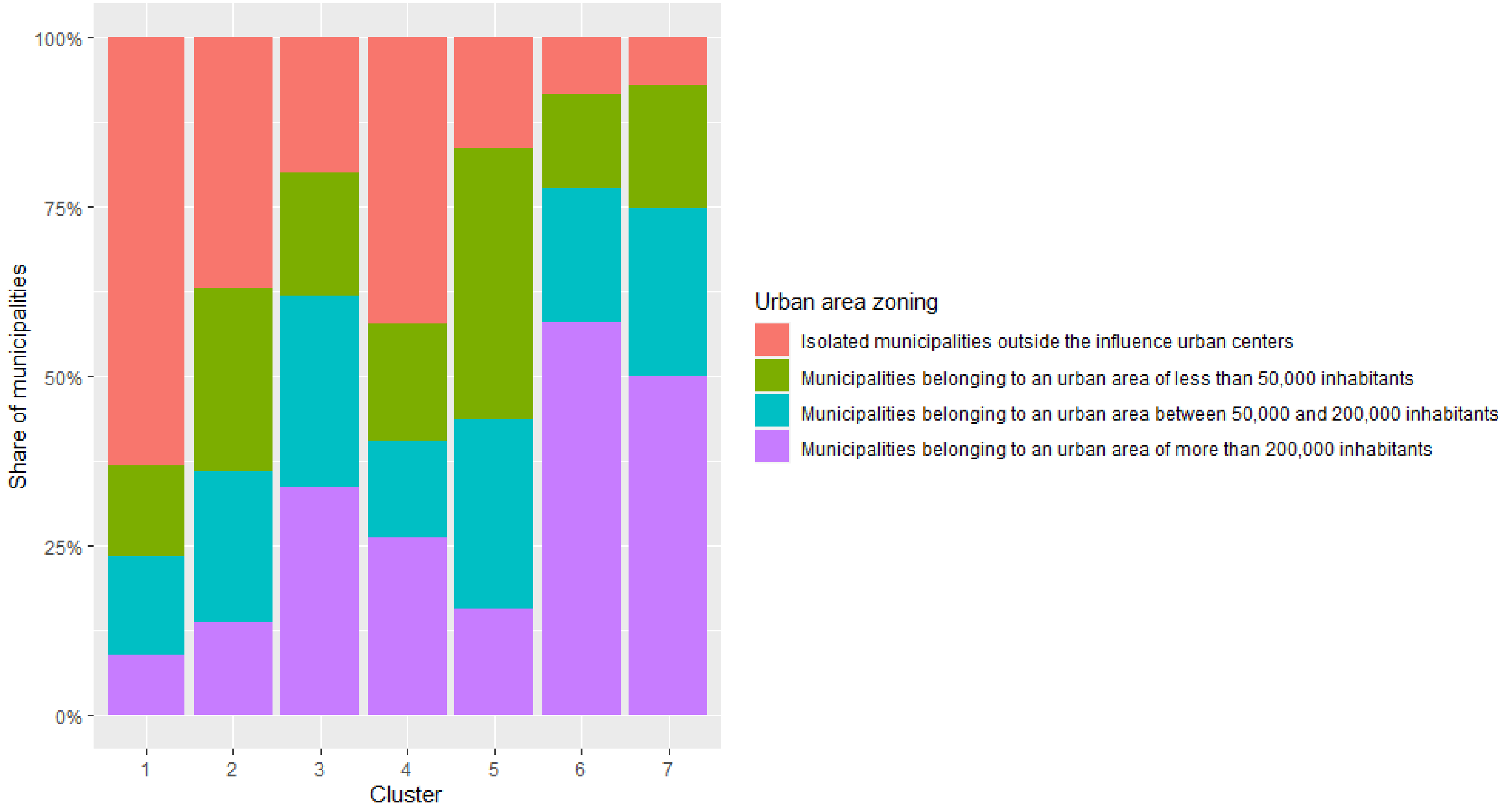

*An urban area is a group of municipalities, in one piece and without enclave, which defines the extent of the influence of a population and employment center on the surrounding municipalities, this influence being measured by the intensity of commuting.

<u>Level of centrality of municipalities by cluster</u>

-

All municipalities included

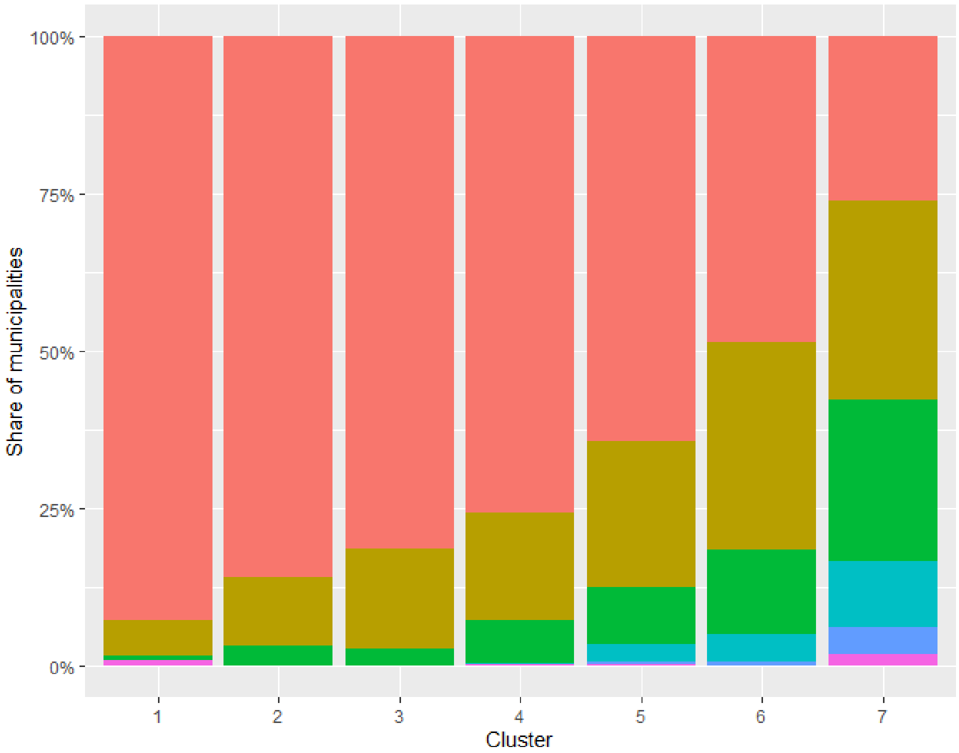

Only for isolated municipalities outside the influence of urban centers

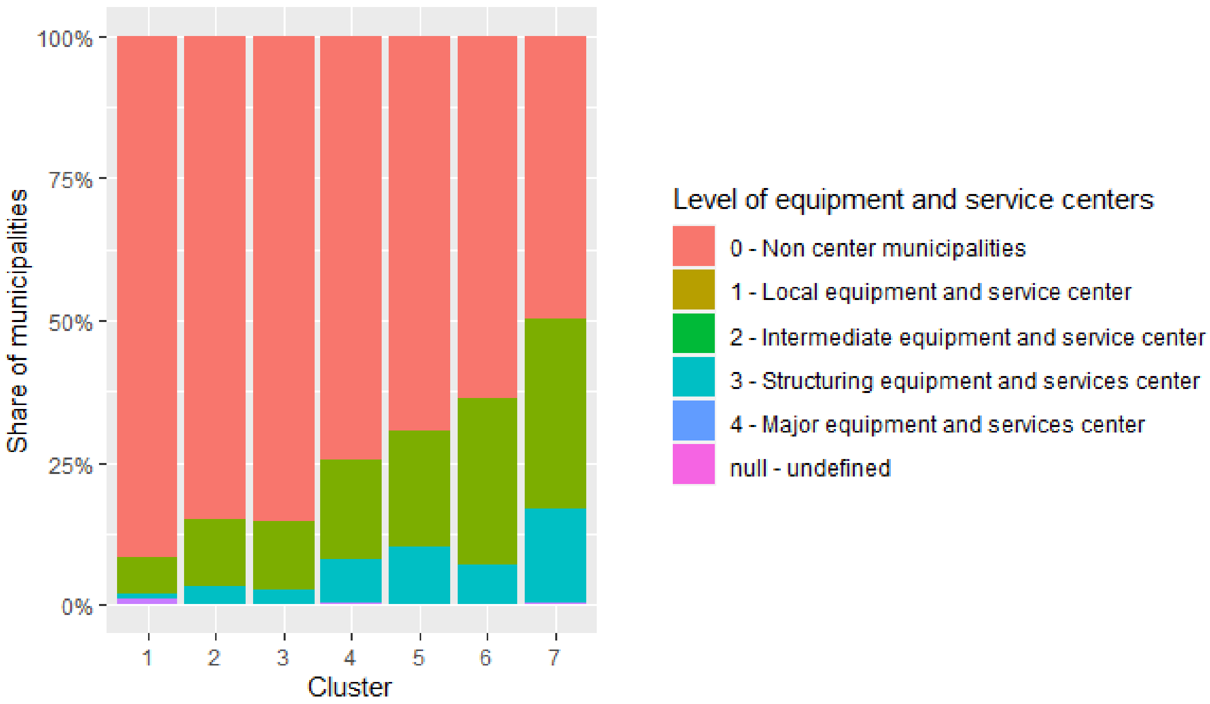

*The levels of equipment and service centers is derived from a study conducted by INRAE-CESAER in partnership with ANCT. The centers are identified based on the diversity of equipment and services present in the municipalities. The greater the level of centrality, the greater the diversity and scarcity of facilities.
